# Fitness level and body composition indices: cross-sectional study among Malaysian adolescent

**DOI:** 10.1186/1471-2458-14-S3-S5

**Published:** 2014-11-24

**Authors:** Redzal Abu Hanifah, Hazreen Abdul Majid, Muhammad Yazid Jalaludin, Nabilla Al-Sadat, Liam J Murray, Marie Cantwell, Tin Tin Su, Azmi Mohamed Nahar

**Affiliations:** 1 Department of Sports Medicine, University Malaya Medical Centre, 59100 Kuala Lumpur, Malaysia; 2 Sports Medicine Unit, Faculty of Medicine, University Malaya, 50603 Kuala Lumpur, Malaysia; 3 Centre for Population Health (CePH), Department of Social and Preventive Medicine, Faculty of Medicine, University Malaya, 50603 Kuala Lumpur, Malaysia; 4 Department of Paediatrics, Faculty of Medicine, University Malaya, 50603 Kuala Lumpur, Malaysia; 5 Centre for Public Health, Queens University Belfast, Royal Victoria Hospital, Grosvenor Road, Belfast, Northern Ireland, UK

**Keywords:** Metabolic risk factors, step test, adiposity parameters, physical fitness, adolescent, Malaysia

## Abstract

**Background:**

The importance of fitness level on the well-being of children and adolescent has long been recognised. The aim of this study was to investigate the fitness level of school-going Malaysian adolescent, and its association with body composition indices.

**Methods:**

1071 healthy secondary school students participated in the fitness assessment for the Malaysian Health and Adolescents Longitudinal Research Team (MyHEART) study. Body composition indices such as body mass index for age, waist circumference and waist height ratio were measured. Fitness level was assessed with Modified Harvard Step Test. Physical Fitness Score was calculated using total time of step test exercise and resting heart rates. Fitness levels were divided into 3 categories - unacceptable, marginally acceptable, and acceptable. Partial correlation analysis was used to determine the association between fitness score and body composition, by controlling age, gender, locality, ethnicity, smoking status and sexual maturation. Multiple regression analysis was conducted to determine which body composition was the strongest predictor for fitness.

**Results:**

43.3% of the participants were categorised into the unacceptable fitness group, 47.1% were considered marginally acceptable, and 9.6% were acceptable. There was a significant moderate inverse association (p < 0.001) between body composition with fitness score (r = -0.360, -0.413 and -0.403 for body mass index for age, waist circumference and waist height ratio, respectively). Waist circumference was the strongest and significant predictor for fitness (ß = -0.318, p = 0.002).

**Conclusion:**

Only 9.6% of the students were fit. There was also an inverse association between body composition and fitness score among apparently healthy adolescents, with waist circumference indicated as the strongest predictor. The low fitness level among the Malaysian adolescent should necessitate the value of healthy lifestyle starting at a young age.

## Background

Physical activity relates to any movement produced by the individual's skeletal muscles that results in energy expenditure [1]. Physical fitness is a set of attributes a person have or achieve, [1] which is linked to the person's capability to do physical activity [2]. Fitness is divided into health and skill related components, with the health component further consists of cardiorespiratory endurance, muscular endurance, muscular strength, and flexibility [1]. An individual who is physically fit has the ability to do daily activities with vigour and alertness, without undue fatigue and still has enough energy to pursue leisure-time activities and prepare for emergencies that ensue [3].

In healthy adults, studies have shown that cardiorespiratory fitness is inversely associated with all-cause mortality and cardiovascular disease [4-6]. Low fitness levels and childhood obesity has been shown to continue into adulthood, with consequent health morbidity like cardiovascular and metabolic diseases [7].In adolescent, most commonly used measurements for adiposity are body mass index (BMI), waist circumference (WC), and waist height ratio (WHtR). BMI [8-10], WC [11-13] and WHtR [14-16] are strong predictors for cardio-metabolic risk factors in the paediatric population.

The previous studies that investigated the association between fitness and body composition indices in adolescent [17-20] used parameters such as BMI or WC. There are no studies that investigate these indices with WHtR, or determine which of the body composition indices in adolescent is the strongest predictor for fitness. This is of clinical interest for the purpose of mass screening and future population based health intervention.

In Malaysia, there are limited community based studies determining the fitness level among adolescent. A few used physical activity questionnaire to determine the physical activity level [21,22]. In this cross sectional study, we investigated the fitness level among Malaysian adolescent using step test. We determined the association between BMI, WC and WHtR to the fitness level and identified which body composition indices is the strongest predictor of fitness.

## Methods

### Study population

The Malaysian Health and Adolescents Longitudinal Research Team (MyHEART) is an on- going prospective longitudinal cohort study in Malaysia. The first phase (MyHEART I 2012) involved Form 1 students (13 years old) from 15 rural and urban secondary schools randomly selected in three states - Perak, Selangor and Kuala Lumpur. The study lasted for three months from March till May 2012.

Study design, sampling methods, data collection protocols and inclusion and exclusion criteria have been reported elsewhere [23]. Ethical approval which complied with the International Conference on Harmonization - Guidelines for Good Clinical Practice (ICH-GCP) and the Declaration of Helsinki was obtained from the Medical Ethics Committee of the University of Malaya Medical Centre, Malaysia (IRB number 896.34).Both written consent from parents and agreement forms from participants were obtained prior to the study.

### Anthropometric and clinical data

The participants completed a standardised form, which socio-demographic data such as date of birth, age, gender, and ethnicity were collected. Systolic and diastolic blood pressures were measured with a stethoscope and mercurial sphygmomanometer (CK-101C, Spirit Medical Co., Taiwan).The participants sit upright with the right upper arm on the table at the level of the heart. They rested for 5 minutes prior to measurement. Pulse rate was obtained with a finger pulse oximeter (Baseline 12-1926 Fingertip Pulse Oximeter, Fabrication Enterprises Inc., USA).

Height was measured using a stadiometer (Seca Portable 217 Seca, UK) and recorded to the nearest 0.1 cm. Weight was measured with a digital electronic weighing scale (Seca 813, Seca, UK) and recorded to the nearest 0.1 kilogram. BMI was calculated by using weight in kilograms divided by the square of height in meters. BMI standard deviation (BMI SD) for age and gender was calculated using the World Health Organisation (WHO) Anthro Software version 3.2.2 for SPSS macro, based on WHO reference 2007 (WHO,Geneva, Switzerland). WC was measured with a non-elastic measuring tape (Seca 201, Seca, UK) mid-point between the lowest rib margin and the iliac crest, and recorded to the nearest millimetre. WHtR was determined by WC in cm divided by height in cm.

Sexual maturation was based on self-reported Tanner staging questionnaire[24]. External genitalia development and pubic hair in boys, and breast development and pubic hair in girls were used for Tanner staging.

### Fitness test

Fitness test was conducted using the modified Harvard Step Test [25,26]. It utilized a 30 cm high step box which was readily available for fitness assessment in all secondary schools in Malaysia. A finger pulse oxymeter was attached to one of the fingers to continuously monitor the pulse rate and oxygen saturation (SpO_2_). With a metronome set at 120 beats per minute, the participants stepped on and off the box with both feet for 5 minutes. Those with heart rates above 200 beats per minute, had difficulty in breathing, SpO_2 _less than 90% or unable to finish, were stopped immediately. Once the participants have completed the step test or were stopped due to the aforementioned reasons, they quickly sit down on the box and rest. Heart rates at 0, 1 and 2 minutes of rest were recorded, as well as total duration of exercise in seconds. The fitness test was conducted under close supervision of a sports physician.

Physical fitness score (PFS) was calculated by the total duration of exercise in seconds × 100 and divided with the sum of three heart rates at 0, 1 and 2 minutes of rest. PFS < 55 is considered poor, 55 - 64 as low average, 65 - 79 as high average, 80 - 89 as good, and > 90 as excellent. The medium score (65-79) was used as a cut- off point to separate the participants of upper and lower PFS, thus <65 is considered unacceptable, 65-79 as marginally acceptable, and >80 as acceptable [25,26].

### Statistical analysis

Test for normality was conducted for continuous variables. Analysis was done separately by gender. Mean ± SD was calculated for both baseline characteristics and exercise values. Independent t-test and chi square test were used where appropriate to compare between gender differences. Partial correlation coefficient was used to examine the direction and strength between body composition indices with PFS, with controlling of factors - age and dummy coded gender, locality (urban/rural), smoking status, Tanner staging and ethnicity. Multiple regression analysis was performed to determine the strongest body composition predictor for PFS, with the same controlling factors stated. All statistical analyses were completed using SPSS version 20 and the level of significance at p < 0.05.

## Results

### Descriptive statistics

1361 students participated in the MyHEART study. However, 285 participants refused to be involved in the exercise test, while five were excluded due to illness or with pre-existing medical condition. Thus, PFS data was available from 1071 participants.

**Figure 1:**
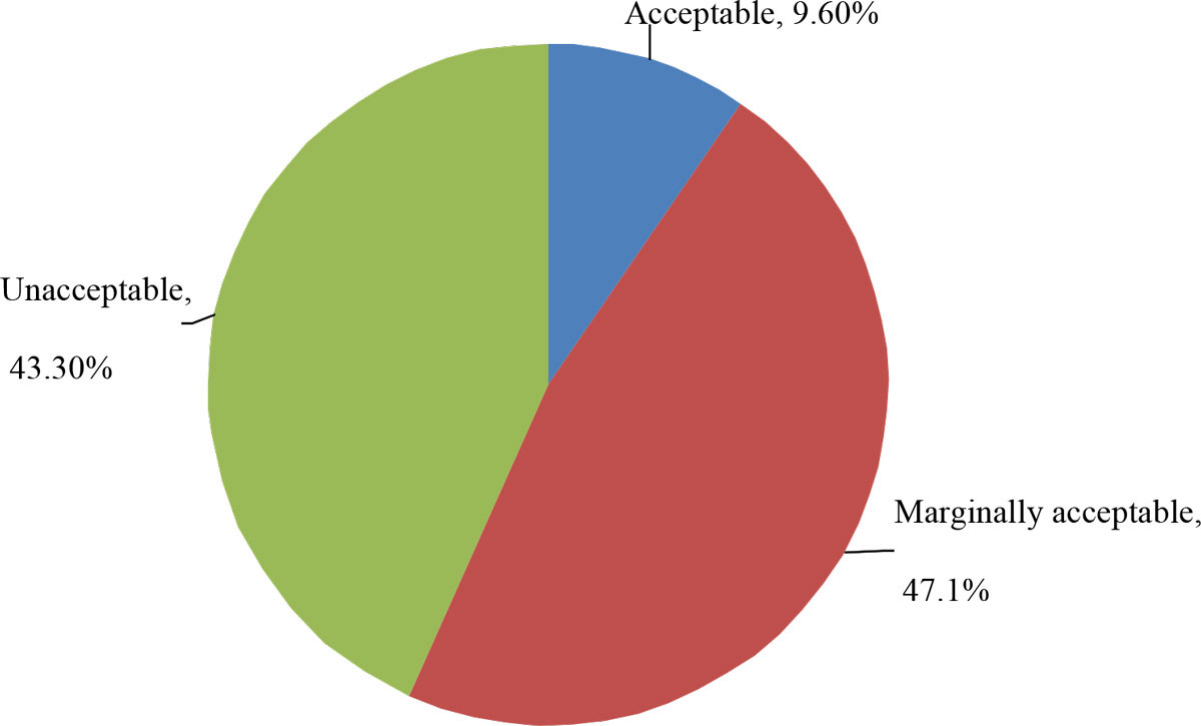
**Physical Fitness Score (PFS) of participants**.

Table 1 and table 2 present the baseline characteristics and exercise parameters of the participants. Boys had a significantly higher WC and WHtR (p < 0.001) compared to girls. No significant difference for BMI and BMI SD in both genders. Boys had a higher mean PFS than girls (71.2 ± 13.1 vs. 61.1 ± 12.5, p < 0.001). When we further categorize the PFS (Figure 1), only 9.6% of the participants were in the acceptable range, with 21.8% of boys and 1.9% of girls. 47.1% were marginally acceptable (55.9% of boys, 41.7% of girls) and 43.3% were in the unacceptable fitness level (22.3% of boys, 56.4% of girls).

**Table 1:** Baseline characteristics of participants.

	Boys	Girls	*p*
n	405	666	-
Ethnicity (%)			0.87
Malay	82.3	83.0	
Chinese	8.2	7.0	
Indian	6.5	7.1	
Others	3.1	2.8	
Tanner staging (%)			<0.001
1	8.2	4.8	
2	30.5	18.2	
3	40.8	48.4	
4	17.7	25.3	
5	2.9	3.4	
Age (years)	12.8 ± 0.3	12.9 ± 0.3	0.024
Pulse rate (beats/min)	85.5 ± 14.0	90.1 ± 13.2	<0.001
SBP (mm/Hg)	110.6 ± 10.5	108.7 ± 11.8	0.006
DBP (mm/Hg)	68.8 ± 10.5	66.7 ± 10.3	0.001
BMI (kg/m^2^)	19.8 ± 5.0	19.7 ± 4.4	0.729
BMI SD	0.17 ± 1.7	0.02 ± 1.4	0.130
WC (cm)	70.5 ± 13.1	67.5 ± 9.9	<0.001
WHtR	0.47 ± 0.1	0.45 ± 0.1	<0.001

Data are mean ± SD or percentage, SBP = Systolic blood pressure, DBP = Diastolic blood pressure, BMI = Body mass index, WC = Waist circumference, WHtR = Waist height ratio.

**Table 2:** Exercise parameters of participants.

	Boys	Girls	*p*
Pulse rate at 0 min rest (beats/min)	171.4 ± 17.8	180.2 ± 15.2	<0.001
Pulse rate at 1 min rest (beats/min)	125.8 ± 19.8	144.4 ± 15.7	<0.001
Pulse rate at 2 min rest (beats/min)	115.0 ± 17.5	131.1 ± 14.6	<0.001
Total duration of exercise (s)	289.8 ± 40.0	277.6 ± 54.2	<0.001
PFS (mean)	71.2 ± 13.1	61.1 ± 12.5	<0.001
PFS (%)			<0.001
Unacceptable	22.3	56.4	
Marginally acceptable	55.9	41.7	
Acceptable	21.8	1.9	

Data are mean ± SD or percentage, PFS= Physical fitness score

### Correlation analysis and multiple linear regression analysis

Correlation analysis in Table 3 showed BMI SD, WC and WHtR were moderately inverse correlated with PFS (r = -0.360, r = -0.413 and r = -0.403 respectively, p < 0.001). The strongest correlation was with WC. Multiple regression analysis in Table 4 showed that WC was the only significant predictor for PFS (ß = -0.318, p = 0.002).

**Table 3:** Correlation coefficient (*r*) between body composition indices and physical fitness score using partial correlation analysis*

Body composition	Physical fitness score	
	***r***	***p***

BMI SD	-0.360	<0.001
WC (cm)	-0.413	<0.001
WHtR	-0.403	<0.001

BMI = Body mass index, WC = Waist circumference, WHtR = Waist height ratio

*Control for age, gender, smoking status, ethnicity, locality and sexual maturity

**Table 4:** Standardised coefficient (ß) between body composition indices and physical fitness score using multiple regression analysis*

Body composition	Physical fitness score	
	**ß**	***p***

BMI SD	-0.007	NS
WC (cm)	-0.318	0.002
WHtR	-0.067	NS

NS = Not significant, BMI = Body mass index, WC = Waist circumference, WHtR = Waist height ratio

*Control for age, gender, smoking status, ethnicity, locality and sexual maturity

## Discussion

### Fitness level

This study showed that only 9.6% of Malaysian adolescent were considered fit (acceptable range) after the step test. The ratio of fitness for girls to boys was more than 1:10 (1.9% and 21.8% respectively). The low level of fitness among the study group emphasizes the need to identify and instil healthy lifestyle choices from young. Other Malaysian studies have shown that between 3%-20% of adolescent had high levels of physical activity, 50%-61.5% were moderately active and 30%-35.3% had low physical activity levels [21,22]. However, these studies were carried out only in schools of one district in a Malaysian state. Moreover, the result was based on physical activity questionnaire, which assessed reported behaviours of the participants, while the current study objectively assessed the fitness level of adolescent.

Our result also showed that boys were fitter than girls. This was in accordance to other adolescent population in Asian countries [27-29], Europe [20,30,31] and North America [32,33]. Gender difference of fitness level could be due to variances in haematological parameters and ventricular chamber sizes [34,35].

This low level of fitness is a global problem. Based on analysis of 50 studies involving 25 million children from 28 countries spanning from 1964-2010, our current children generation were 15% less fit than when their parents were at their age [36]. Low fitness level in the paediatric population is associated with higher risk of a cluster of cardio-metabolic risk factors [31,37-39]. Various studies have shown the benefits of intervention programs in improving children's and adolescent's health status i.e. reduce body fat [40-43], improve metabolic syndrome score [44,45], positive effect on blood pressure [46,47], increase bone density [48,49] and academic performances [50,51].

### Body composition indices and fitness

This study showed that BMI SD, WC and WHtR were all inversely correlated with fitness level. Our result was similar with findings from other studies [17-20]. Another study also showed that visceral adipose tissue measured with abdomen MRI was inversely correlated with aerobic fitness [52].Moreover, our study also investigated the correlation with WHtR, which is also considered as a marker of intra-abdominal adiposity in children and adolescent [53]. WHtR was shown to be a good predictor of cardio-metabolic risk factors [14-16].

WC was the strongest predictor to fitness in our findings. This reiterates the importance of WC as a measurement tool of health status for adolescent. It is a surrogate marker for intra-abdominal adiposity [54], and a strong predictor for cardio-metabolic risk factors in children and adolescent [11-13]. The actual relation of WC on cardio-metabolic risk factors is still unclear. A few possible hypotheses have been proposed. Intra-abdominal adiposity is an indicator of impaired energy storage regulation, which leads to an excess of fat collection in the liver, and subsequent compromised liver function of fat regulation. Thus, increased intra-hepatic fat storage consequently leads to dyslipidaemia and insulin resistance [55]. Another probable way is the release of free fatty acids through lipolysis of omentum and mesenteric adipocytes, which in turn triggers insulin resistance and hypercholesterolemia [56].

Other studies have investigated the influence of fitness on adiposity indices using multiple linear regression analysis. In one study, fitness was determined to be an independent predictor WC, visceral and abdominal subcutaneous adipose tissue after adjusting factors of age, gender, sexual maturity and BMI [17]. Another showed that VO_2_max, a marker of fitness, was inversely associated with both BMI and WC after controlling factors of age, sexual maturity and active commuting to school [19]. Interestingly, it showed that adolescent with low fitness level were associated with increased 5.6 cm and 2.9 cm of waist circumference in boys and girls respectively, as compared to those in the higher fitness level. A third study in Spain revealed cardiorespiratory fitness was the strongest predictor of BMI, sum of skinfold thickness and subcutaneous truncal fat, as compared to physical activity level [57]. All these warrant the importance of active intervention and healthy lifestyle to start at a young age.

American College of Sports Medicine recommends daily moderate to vigorous intensity aerobic exercise for ≥60 minutes per day for children [2]. Three hours per week sports participation has shown to reduce body fat and increase fitness level among boys [58]. A VO_2_max of 45 ml/kg/min for boys and 40.7 ml/kg/min for girls may be the minimum level to limit accumulation of intra-abdominal adiposity [19].

School based intervention programs in Malaysia showed positive effects on fitness and flexibility [59]. However, these studies included additional physical activity programs on top of the compulsory physical education classes. Future studies are needed to investigate the implication of physical education classes on Malaysian adolescent's health status.

### Strength and limitation

The strength of our study is the large sample of participants. To our knowledge, this is the first large cross-sectional study in Malaysia to determine the fitness level among school-going adolescent. This study utilized modified Harvard step test as a tool for fitness assessment, which does not require large space to conduct, minimal equipment and expertise, and a short duration of time to complete (less than 10 minutes). Further studies are needed to compare this step test with other fitness protocols.

There are limitations to this study. Cross-sectional design cannot capture cause-effect relationship. The cohort sample are of the same age group, thus this does not reflect the whole adolescent population. More than 80% of participants were Malays, which do not reflect the racial composition of Malaysian adolescent. However this study is still of importance since it is one of a few that assessed fitness level. Our next step would be in MyHEART II 2014 where a follow-up of the same participants would be conducted, thus a longitudinal association between body composition indices with fitness and changes in fitness level could be determined.

## Conclusions

This study showed only 9.6% of Malaysian adolescent were categorised as fit, with ratio of girls to boys of 1:10. BMI SD, WC and WHtR were inversely correlated with fitness, and WC was the strongest predictor for fitness. The importance of being physically active and fit needs to be emphasized by all stake-holders and nurture healthy lifestyle choices for the young.

## List of abbreviations used

MyHEART: Malaysian Health and Adolescents Longitudinal Research Team; BMI: Body mass index; WC: Waist circumference; WHtR: Waist height ratio; ICH-GCP: International conference on harmonization - guidelines for good clinical practice; SpO_2_: Oxygen saturation; SD: Standard deviation; WHO: World health organization; PFS: Physical fitness score.

## Competing interests

The authors declare that they have no competing interests.

## Authors' contributions

All authors contribute to study design, revising and improving the manuscript; RAH, NAAM, MYJ, HAM and TTS were involved in field work and data collection; RAH, MNAM, HAM, LM, MC and TTS participated in analysis and interpretation of data; RAH drafted the manuscript.
